# Mitotherapy: Unraveling a Promising Treatment for Disorders of the Central Nervous System and Other Systemic Conditions

**DOI:** 10.3390/cells10071827

**Published:** 2021-07-20

**Authors:** Gabriel Nascimento-dos-Santos, Eduardo de-Souza-Ferreira, Rafael Linden, Antonio Galina, Hilda Petrs-Silva

**Affiliations:** 1 Instituto de Biofísica Carlos Chagas Filho, Universidade Federal do Rio de Janeiro, Rio de Janeiro 21941-901, Brazil; gabriel.nascimento@biof.ufrj.br (G.N.-d.-S.); rlinden@biof.ufrj.br (R.L.); 2 Instituto de Bioquímica Médica Leopoldo de Meis, Universidade Federal do Rio de Janeiro, Rio de Janeiro 21941-901, Brazil; esferreira@bioqmed.ufrj.br

**Keywords:** mitotherapy, mitochondria, therapy, neurodegeneration, central nervous system

## Abstract

Mitochondria are key players of aerobic respiration and the production of adenosine triphosphate and constitute the energetic core of eukaryotic cells. Furthermore, cells rely upon mitochondria homeostasis, the disruption of which is reported in pathological processes such as liver hepatotoxicity, cancer, muscular dystrophy, chronic inflammation, as well as in neurological conditions including Alzheimer’s disease, schizophrenia, depression, ischemia and glaucoma. In addition to the well-known spontaneous cell-to-cell transfer of mitochondria, a therapeutic potential of the transplant of isolated, metabolically active mitochondria has been demonstrated in several in vitro and in vivo experimental models of disease. This review explores the striking outcomes achieved by mitotherapy thus far, and the most relevant underlying data regarding isolated mitochondria transplantation, including mechanisms of mitochondria intake, the balance between administration and therapy effectiveness, the relevance of mitochondrial source and purity and the mechanisms by which mitotherapy is gaining ground as a promising therapeutic approach.

## 1. Mitotherapy: A Primer

Despite the early recognized relevance of mitochondria for cell energetics and homeostasis, there was little or no information regarding mitochondria’s dynamism and transfer feasibility between cells. It was only in 1982 that Clark and Shay demonstrated that isolated mitochondria carrying a mutation for chloramphenicol and efrapeptin resistance in its genome, naturally transferred the antibiotic resistances to sensitive cells by simple incubation [1]. Later, evidence was shown that the microinjection of isolated human mitochondria from chloramphenicol resistant cell lines effectively induced antibiotic resistance in human sensitive cells [2]. It was also shown that isolated mitochondria could be inserted into fertilized zygotes by microinjection [3]. These were the first demonstrations of the feasibility of isolated mitochondria to be incorporated and promote modifications in foreign cells.

Still, it took over two decades since Clark and Shay findings for a follow up demonstration of mitochondrial spontaneous transfer. In 2006, Spees and colleagues reported the first evidence supporting the horizontal (cell-to-cell) transfer of mitochondria between mammalian cells. The co-culturing of A549 mutated and depleted cells with human mesenchymal stem cells (MSCs) successfully rescued the mitochondrial function in the former, as shown by the increase in ATP content, membrane potential and oxygen consumption [4]. Corroborating this finding, isolated mitochondria were also spontaneously taken up by A549 p^0^ cells, successfully restoring mitochondrial respiration [5].

In the last two decades, several studies showed an in vitro relocation of mitochondria, either by horizontal transfer (for more information, please see Berridge and colleagues [6]) or an incorporation of isolated organelles by other cells. Those studies confirmed the dynamism of mitochondria and set the ground for the current literature on mitotherapy, i.e., the treatments with isolated mitochondria and the main focus of this review.

## 2. Mitotherapy in Animal Models of Disease

Based on the above findings, the first study of the potential of mitotherapy in an animal model was published by McCully and colleagues in 2009 [7]. Respiration-competent mitochondria, isolated from healthy cardiac tissue were injected directly in the ischemic area of rabbit hearts during early reperfusion, which successfully enhanced myocardial functional recovery and cell viability (Table 1). Corroborating this finding, a few years later the same group showed that the autologous local transplantation of isolated mitochondria protected cardiomyocytes from ischemia-reperfusion injury [8]. In the following years, the McCully group showed the effectiveness of mitotherapy in an ischemic rabbit’s heart following a less invasive perfusion of mitochondria via the coronary artery [9], as well as in a porcine model of ischemia/reperfusion [10] and in prolonged cold ischemia after murine heart transplantation [11]. Other studies reported that an infusion of preischemic mitochondria in the coronary artery reduced the infarct size and improved heart function both in pigs [12] and in diabetic rats [13]. Thus, these findings indicated that mitotherapy may be effective against cardiomyocyte-related injuries.

Studies of the in vivo models of liver insults indicate that transplanted mitochondria may also contribute to metabolic recovery of hepatocytes. For example, an intrasplenic infusion of isolated mitochondria attenuated oxidative stress, cell death and the serum levels of liver enzymes in a rat liver model ischemia-reperfusion [14]. The systemic administration of isolated mitochondria also restored hepatic function by mitigating serum aminotransferase activity, cholesterol levels, lipid accumulation and oxidative stress in a mouse model of non-alcoholic fatty liver disease [15]. Similar efficiency was reported for a mouse model of acetaminophen-induced liver injury [16], thus suggesting mitochondria transplantation as a feasible therapy approach for liver-related diseases.

In the field of respiratory diseases, an intratracheal injection of mitochondria reversed airway hyperresponsiveness to acetylcholine in rats [17]. The transplantation of mitochondria via pulmonary artery delivery or aerosol nebulization also improved dynamic compliance and inspiratory capacity and decreased tissue injury after ischemia-reperfusion in mice [18]. Further, liver-isolated mitochondria successfully inhibited lung tumor growth in mice by reducing glycolysis and oxidative stress [19].

In parallel with the studies above, evidence was gathered that mitotherapy may also be protective in the central nervous system (CNS), as shown in the next section.

## 3. Mitotherapy in the Central Nervous System

Diseases of the CNS are often terminal due to the intrinsic inability of central neurons to regenerate and its non-permissive microenvironment. Among new therapeutic approaches to overcome these impediments, several studies examined the potential of mitochondria transplantation in neurological disorders (Table 2).

In the first such study, the mitochondria derived from hamsters attenuated the area of brain infarction and neuronal death, and restored motor performance in a stroke model of middle cerebral artery occlusion (MCAO) in rats, either via local intracerebral or systemic intraocular-arterial administration. In addition, the transient proliferation of microglia and astrocytes suggested a role of immune response to mitotherapy [21]. In subsequent studies, the transplantation of autologous mitochondria via intracerebroventricular (ICV) administration promoted neuroprotection, reduced brain infarct size, ameliorated functional deficits and induced neurogenesis at the boundary of an ischemic area in an MCAO model, but in contrast with the previous study, autologous transplantation reduced reactive astrogliosis [22]. Together, these data suggest that mitotherapy may prevent the death of central neurons and neurologic impairment following ischemic stress.

A few studies also tested the efficacy of mitotherapy in models of Parkinson’s disease (PD), a prevalent and disabling neurodegenerative condition characterized by progressive motor as well as eventual cognitive dysfunction. There is strong evidence of mitochondrial dysfunction in PD, manifested by a reduced bioenergetic potential, the disruption of redox homeostasis and an increased susceptibility to excitotoxic neuronal death. In a well-established rat model of PD produced by the administration of the neurotoxin 6-hydroxydopamine, an injection of mitochondria in the medial forebrain bundle successfully attenuated the oxidative damage and degeneration of dopaminergic neurons and improved locomotion [23]. In another study, the intravenous administration of mitochondria prevented the progress of experimental PD in mice treated with the neurotoxin MPTP, by hampering neuronal death and attenuating damage by reactive oxygen species. Consistent with the previous data, the treatment improved the behavioral symptoms of the PD in both pole and rotarod tests. Mitotherapy also resulted in a longer swimming time in healthy mice, consistent with the hypothesis that mitochondria can modulate bioenergetic homeostasis and increase mouse endurance [24]. These data suggest that mitotherapy may attenuate the degenerative progression in PD and demonstrate that the intravenous administration of mitochondria leads to widespread distribution to the brain, thus suggesting the feasibility of mitochondrial transplantation to the CNS.

Mitotherapy also showed promising results in experimental models of schizophrenia (SZ) and Alzheimer’s disease (AD). Several mitochondrial dysfunctions were reported in SZ, including alterations of proteome, disruption in the cellular respiration and dissipation of membrane potential (Δψm) [25,26]. In a rodent model of SZ, an injection of mitochondria into the prefrontal cortex of young rats successfully prevented the loss of brain Δψm and attention deficit in adulthood [27]. Mitochondria dysfunction is also involved with damage to neurons by amyloid-β (Aβ) and phosphorylated tau protein, both hallmarks of AD [25,28]. In an AD mouse model produced by the intracerebroventricular injection of Aβ peptide, the intravenous administration of mitochondria derived from HeLa cells attenuated neuronal loss and reactive gliosis, and reverted deficits of fear memory in a freezing test; short-term memory in a y-maze test; long-term non-associative memory in the open field; and working, spatial learning and cognitive ability in a radial water maze [29]. Thus, mitochondria transplantation may eventually be useful as a therapeutic approach to both SZ and AD, correcting brain pathology and restoring cognitive deficits.

The impairment in mitochondria homeostasis is observed in psychiatric disorders as well. In a lipopolysaccharide (LPS)-induced model of depression, an intravenous administration of mitochondria reduced the period of immobility in the forced swimming and tail suspension tests, improved sucrose preference and exploratory behavior in the open field test, attenuated astrogliosis and microglia activation, restored ATP production and promoted neurogenesis [30]. These data suggest that the transplantation of mitochondria led to antidepressant-like effects, modulated the brain immune response, mitochondria bioenergetics and adult hippocampal neurogenesis. The systemic administration of mitochondria also increased the forced swimming score and decreased latency on water maze tests in aged mice, although the treatment did not match the aging effects to young mouse scores [31].

Unlike the peripheral nervous system, central neurons lack the ability to successfully regenerate axons toward their targets after injury. Despite several efforts to overcome the inhibitory microenvironment and limited intrinsic growth capacity, there are no definitive therapeutic approaches available. In the pursuit of new strategies, mitochondria dynamics have been constantly related to retinal ganglion cell (RGC) viability and axonal regeneration in several models [32,33,34]. For instance, in injured axons of *C. elegans*, mitochondria density increases and correlates with regeneration [35], which requires proper energy production [36]. Mitochondria were also reported as necessary to prevent axonal degeneration after injury, and genetic manipulation of mitochondrial transport enhances regeneration by the translocation of mitochondria to injured axons, rescuing energy deficits in rats [35,37]. Thus, mitochondria dynamics appear to be a key to induce axonal regeneration in the CNS after injury. Early studies of mitochondria supplement in central neuron regeneration were conducted in vitro. Isolated mitochondria restored the membrane potential of injured hippocampal neurons and significantly increased neurite outgrowth [38]. Furthermore, mitotherapy improved hindlimb motor function in the Basso–Beattie–Bresnahan score in a rat model of spinal cord ischemia [39]. In contrast, another study did not find an enhancement of either hindlimb sensitivity or motor function scores in long-term evaluation after the treatment of a spinal cord injury [40]. There are, however, important differences between the above-described experiments that may explain the opposed results, such as distinct experimental models and time after surgery.

Our own group addressed the regenerative potential of mitochondria transplantation in an experimental model of glaucoma based on optic nerve crush in rats [41]. An intravitreally injected single bolus of liver-isolated mitochondria successfully promoted short-term neuroprotection (14 days) to RGC and modulated retinal oxidative metabolism. Importantly, mitochondria also increased the number of axons extending ahead of the injury site in a long-term period (28 days). It is yet to be tested, however, if the intravenous administration of mitochondria would lead to the same results, which would be an advantage due to the chronic nature of glaucoma. Nonetheless, these data suggest that mitotherapy is a promising approach to counteract the inability of axon regeneration in the CNS. In turn, the in vivo findings encourage further investigation of the potential of mitochondrial transplantation as a tool to control neurodegeneration in neurological disorders.

## 4. Route of Administration and Its Relevance for Mitotherapy

An important matter for mitotherapy is the route of delivery. Most studies have either administered directly into the target tissue or at the respective artery, proximal to the area of interest, while others have used a systemic route.

Systemic administration was consistently performed in several studies [15,16,19,24,31]. Once applied in the tail vein of rodents, labelled mitochondria were spotted in many different organs, and following transplantation into the rat spleen, mitochondria were detected in the liver [14]. Unexpectedly, despite the highly selective CNS blood–brain barrier (BBB), mitochondria administered by systemic intra-arterial injection reached ischemic rat brains [21], demonstrating that the organelles may cross the BBB and provide neuroprotection. Corroborating this hypothesis, X. Shi and colleagues first demonstrated using confocal imaging that mitochondria injected in mouse tail veins effectively reached the brain [24]. The remarkable ability of mitochondria to cross the BBB opens several opportunities to treat CNS diseases in less invasive ways.

Other groups have chosen a more direct way to deliver mitochondria to the tissue of interest. The group of James McCully, who mainly focus on cardiac tissue, have provided important advances for the in loco administration of mitochondria. In their studies, they either injected mitochondria directly into the cardiac tissue [7,8,9,10] or administered isolated mitochondria in the coronary artery to deliver the organelles to the heart [9,11,12,13,42,43], and found less diffused mitochondria when applied intratissue [9]. The group also directly injected mitochondria into ischemic/reperfused skeletal muscle [18] and into injured lung models [40]. Interestingly, in ischemia-reperfusion injured lungs, the authors compared a vascular administration by pulmonary artery delivery with aerosol nebulization. Both administrations improved lung mechanics and decreased lung tissue injury, setting the ground for the establishment of new less invasive techniques for the administration of mitochondria. Furthermore, in the CNS, mitochondria were also directly injected into the mediolateral gray matter of rats that underwent a spinal cord injury [40], into the intra-prefrontal cortex in a model of schizophrenia [27], intraventricularly after ischemic injury [22] and into the ischemic striatum after a stroke [21]. Our group treated rats that underwent an optic nerve crush by intravitreal injection, restricting the isolated mitochondria within the humor vitreous and allowing the organelles available to be taken up by RGC, which provided neuroprotection and neuroregeneration [41].

Some sort of tropism appears to regulate the internalization of mitochondria, since experiments with no tissue injury displayed exogenous mitochondria dispersed in the organism [15,16,24], while the injured tissues were more susceptible to mitochondrial internalization. For instance, in rats, the intravenously administered mitochondria reached a higher concentration in the melanoma lungs than in the healthy ones [19]. The rats that underwent spinal cord ischemia displayed an accumulation of labeled-exogenous mitochondria around the dorsal region, while sham animals had diffused labelling at the left upper abdominal quadrants, suggesting a selective distribution of mitochondria in the ischemic tissues [39]. This could be due to the increased permeability of the tissue near the lesion site, which would allow for a faster dissemination of the transplanted organelle to the target region. However, since immune cells are attracted to the damaged tissue, we cannot rule out the possibility that mitotherapy provided a boost to regenerative effects. In summary, either the systemic or the local administration of mitochondria showed evidence of effectiveness and the route of delivery for the forthcoming clinical interventions may rely on the pathology particularities.

## 5. The Suitability of Mitochondria Source

The source of mitochondria for mitotherapy is critical and depends on the ease to obtain the organelles, the age of the tissue, the donor’s health conditions, the metabolic characteristics of the organ of origin, as well as histocompatibility. Our group chose the liver as the donor of mitochondria based on the high density of the organelle, easy access to the tissue, high regenerative potential and relatively high oxidative phosphorylation coupling index [41].

The variations of the bioenergetic characteristics of mitochondria among organs have already been reported such as the high membrane potential observed in muscle mitochondria, although muscle, brain, brown and white adipose tissue showed no significant differences in mitochondria number [44,45]. Notably, a recent study suggested that the age of the donor tissue may be relevant [19], since mitochondria from young and healthy mice showed better performance to constrain tumor cell proliferation when compared to aged mitochondria, which may be related to the higher membrane potential and antioxidative capacity. The mitochondria from diabetic rats also contain less ATP than control rats after warm global ischemia [13]. These reports indicate that the biological conditions of the donor tissue that affect the bioenergetic performance of the organelles may account for their therapeutic potential. These findings imply that autologous transplantation may not be the best choice for mitotherapy in several diseases, since their therapeutic potential may be hampered by the health of the patient.

The relevance of histocompatibility for the transplantation of mitochondria is still uncertain. In vitro analysis indicated that resistance from the host cells did not happen following the insertion of mouse mitochondria in a human cell line, suggesting that there are strong limits for cells to incorporate exogenous mitochondria [1]. In contrast, the first evidence of a successfully xenogeneic transplantation demonstrated that mitochondria isolated from the Algerian mouse, *Mus spretus*, induced transformation in *M. m. domesticus* mice zygotes after microinjection [3]. Consistent with the latter findings, incubation with murine mitochondria improved the respiratory function in human p^0^ cells [5].

In the first study in adult animals, the mitochondria isolated from a hamster cell line successfully provided neural protection against ischemic stress in rats [21]. Further, both allogeneic and xenogeneic mitochondria improved locomotive activity and attenuated the death of dopaminergic neurons in a rat model of Parkinson’s disease. Nevertheless, xenogeneic transplant was less efficient than allogeneic in the long term [23], but neither syngeneic nor allogeneic injections led to alloreactivity, allorecognition or damage-associated molecular patterns (DAMPs)-dependent reactions [46].

Taken together, the data indicate that the source of mitochondria is relevant in certain scenarios. However, since tissue-matching, unmatching, autologous, allogeneic and xenogeneic transplants induce cell/tissue protection in various models, the choice in each case may be mainly based on the ease of mitochondria isolation.

## 6. Relevance of Mitochondria Purity and Function

Another important issue is the quality of the isolated mitochondria, which we define based on its functionality: a functional mitochondrion phosphorylates ADP in vitro and displays high oxidative coupling factor, parameters that can be easily checked with the use of a high-definition oximeter [41].

Furthermore, a protocol is recommended to control organelle integrity either using cytochrome C (CytC) on a respiration analysis or performing activity assays of specific enzymes of the mitochondrial matrix. A high-quality mitochondrion must not have its respiration highly induced with CytC; therefore, a threshold must be adopted: we recommend no higher than a 15% increase in oxygen consumption. In addition, no activity of matrix enzymes should be detectable in the supernatant of the organelles. Other experiments can give precise information of the quality and purity of the organelle, but those described above are fast, have low space requirements and can be easily applied to the clinic.

In a series of experiments with either intact or disrupted organelles and with purified components from mitochondria as ATP and DNA, McCully and colleagues showed evidence that the therapeutic effects of mitochondria depend on the functional organelle [7]. Corroborating these data, the pharmacological disruption of oxidative phosphorylation hampered the in vitro benefits of mitochondria [21]. To test whether mitochondria must preserve their ability to perform oxidative phosphorylation as an intact organelle, we compared the therapeutic outcome of the high-quality functional mitochondria with osmotically lysed organelles. Our results clearly demonstrated that the protective and regenerative capacity of mitotherapy on both retinal ganglion cells and the optic nerve relied on functional mitochondria, although we found the short-term regenerative capacity of disrupted organelles [41]. Therefore, although certain mitochondrial components may exert beneficial effects, the data strongly suggest that functional mitochondria are mandatory for successful mitotherapy.

The data above support the relevance of the isolation procedure. However, depending on the donor tissue, the procedure can be too costing to allow its therapeutic use. Soft tissues, such as the brain and liver, are easily homogenized and do not require digestive or rough disruptive processes. On the other hand, tissues such as skeletal muscle require more steps and the isolation may take longer. To overcome this issue, the group of McCully developed an optimized protocol for the purification of skeletal muscle mitochondria [10,47].

Overall, the procedures must be oriented to the acquisition of the highest possible number of mitochondria as quickly as possible to guarantee the quality, purity and sterility of the organelles.

## 7. Mechanisms of Mitochondria Internalization

The precise mechanism of organelle internalization in mitotherapy is still controversial. Despite the lack of scientific data regarding free mitochondria internalization, it is likely that some of the known mechanisms described for horizontal transfer may occur in mitotherapy as well.

**Figure 1:**
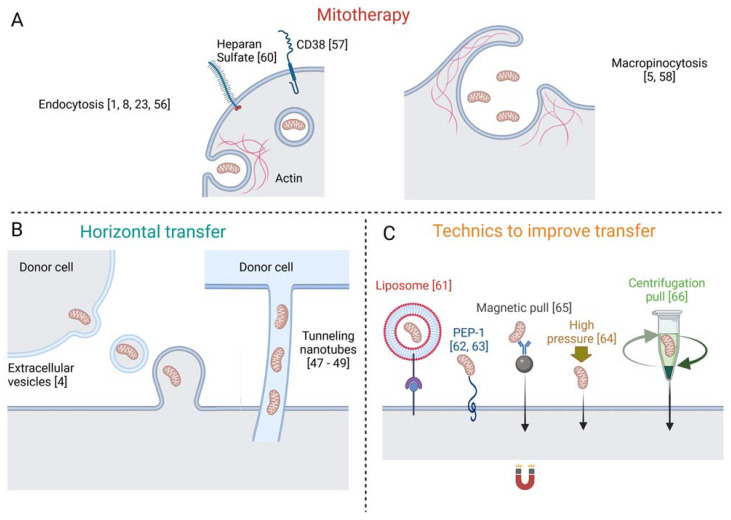
Reported mechanisms and relevant references of mitochondria internalization. Representation of the mechanisms of mitochondria internalization described in mitotherapy (**A**). Mechanisms involved in horizontal transfer of mitochondria (**B**). Technics developed to improve exogenous mitochondria internalization (**C**).

Human MSCs containing healthy mitochondria restored the bioenergetics of A549 cells with deleted or depleted mitochondrial DNA, by transferring healthy mitochondria [4], and the authors suggested that mitochondria take up occurred through extracellular vesicles containing free mitochondria (Figure 1). Additionally, in vitro, labeled mitochondria were transferred from epithelial progenitor cells to cardiomyocytes by tunneling nanotubes [48]. Corroborating the later finding, nanotubes were shown to be critical for the transfer of mitochondria from human MSCs to rat cardiomyocytes [49], and for the rescue of cardiomyoblasts that underwent ischemia by in vitro oxygen and glucose deprivation [50] (Figure 1).

Interestingly, the mitochondria with damaged DNA were unable to be transferred between cells, which suggests a selective process to protect the cell from receiving pathogenic or dysfunctional mitochondria [51]. Yet, an in vivo experiment showed that a canine transmissible venereal tumor acquired healthy mitochondria from the neighboring healthy cells [52]. Mitochondria may also be transferred from astrocytes to neurons, as observed in the brains of the mice that underwent a stroke [53]. An intriguing mechanism was described in which the mitochondria from one cell is transferred or internalized by another cell to be destroyed, characterizing the process of transmitophagy. In the mouse optic nerve head, neuronal mitochondria were degraded by lysosomes of adjacent glia [54], indicating an endogenous neuron-to-astrocyte transport of mitochondria for degradation. Similarly, cardiomyocytes are also able to delivery damaged mitochondria for further degradation by nearby macrophages [55]. This process has been explored in a PD model in which degenerating dopaminergic neurons, incapable of processing mitophagy, accumulated the damaged mitochondria in spheroid-like structures [56]. These mitochondria were primed to undergo mitophagy but lacked the contact proteins that are responsible to link them to the autophagosome. However, astrocytic processes contacted the spheroid and finished the removal of the defective neuronal mitochondria. Mitotherapy also improved the intercellular mitochondrial quality control mechanism and delayed the progression of PD. Exogenous functional mitochondria acted as a rapid repository for the neurons subjected to energetic failure due to the accumulation of damaged mitochondria while boosting astrocytic bioenergetics and increasing transmitophagy turnover. This mechanism may explain how cells that fail mitophagy would benefit from mitotherapy, since the active recycle of damaged organelles would be performed by auxiliary cells, such as astrocyte or macrophages, thus overcoming disturbances in cellular mitochondrial quality control. However, it must be better explored if such a mechanism is present in other tissues.

Regarding specific mechanisms of the internalization of mitochondria, back in 1982, it was already suggested by Clark and Shay that endocytosis was the mechanism involved in the incorporation of free mitochondria [1], and further studies strengthened this hypothesis (Figure 1). McCully and colleagues demonstrated that blocking actin polymerization with Cytochalasin D reduced mitochondrial internalization in cardiomyocytes, strongly suggesting that the mechanism is actin-dependent [57]. Transmission electron microscopy (TEM) detected free mitochondria in vitro, in a process of endocytic engulfment near IPS-derived cardiomyocytes [9]. Further, systemically injected isolated mitochondria were found within a wide range of tissues but not inside mature erythrocytes, which do not perform endocytosis [24]. Still, other mechanisms cannot be excluded, because erythrocytes do not require mitochondria and may selectively reject exogenous mitochondria. In contrast, mouse hepatocytes internalized the isolated mitochondria by endocytosis, as seen by TEM, and glioma cells also internalized the free mitochondria by endocytosis in a CD38-NAD^+^-cADPR-Ca^+2^ signaling pathway [58]. In addition, macropinocytosis, a known subtype of endocytosis, was also proposed as a possible mechanism. Time-lapse fluorescent microscopy revealed the engulfment of mitochondria attached to the surface of rat H9c2 cells [59], which was inhibited by treatment with EIPA, an inhibitor of macropinocytosis. Furthermore, the mitochondria-dependent transformation of the cells was inhibited by pre-treatment with the specific inhibitors of macropinocytosis, Amiloride and EIPA, in a culture of HepG2 [60]. Heparan sulphate proteoglycans were also involved in this process and, recently, a genome-wide CRISPR-cas9 knockout screen showed that the biosynthetic pathway of heparan sulfate was required for macrophages to internalize the free mitochondria [61].

As for clinical applications, several techniques have been used to improve mitochondria transfer, such as the mediation of liposomes [62], membrane penetrating peptide [63,64], application of high pressure [65], magnetic beads [66] and simple centrifugation [67]. Notwithstanding, as commented above, several studies provided evidence that cells are self-sufficient to incorporate isolated mitochondria.

Therefore, evidence is strong that the endocytic mechanism may drive free mitochondria internalization by tissues at large, although tissue-specific factors cannot be excluded, and, as discussed in the beginning of this section, the well-established data regarding the horizontal mitochondrial transfer may be relevant for new strategies of the internalization process in mitotherapy. Still, the mechanisms of free mitochondria internalization must be better understood to further improve mitotherapy’s application.

## 8. Current Scenario of Mitotherapy

A compelling question regarding mitotherapy is how the organelles can induce modification in the target tissue to promote the reported results. Despite the promise of mitotherapy, the mechanisms of mitochondrial effects remain elusive. In this session we addressed both this issue and the clinical advances of mitotherapy.

Of prime concern is whether mitochondria must enter the target cell. Despite the initial evidence from photomicrographs that the internalization of exogenous mitochondria was required for the in vitro gain of antibiotic resistance [1] and respiratory restoration [5], early observations showed that positive therapeutic results were found, while mitochondria were only spotted within the interfibrillar space of myocytes [7]. Similar evidence was gathered of the mitochondria-dependent maintenance of acute bioenergetics in an injured spinal cord, whereas exogenous organelles were not found within the neurons [40].

In contrast, a subsequent publication showed fluorescent-labeled mitochondria within myocytes, even though most transplanted organelles remained in the interfibrillar space [8]. Notably the proportion of ~3–7% of internalized mitochondria in that study is consistent with the achievement of bioenergetic, neuroprotective and pro regenerative effects in our study of the RGC layer of the retina, where only a few neurons contained fluorescent-labeled mitochondria within the cytoplasm at 24 h after injection [41]. In the case of our study, we cannot exclude the possibility that the reported effects depend on mitochondria in the extracellular medium, a hypothesis supported by the results reported for patients of subarachnoid hemorrhage, of a correlation of good clinical recovery with extracellular mitochondria in the cerebrospinal fluid that showed higher membrane potential [68].

An additional issue is the dose–response of mitochondria for therapy efficiency. Here, the concentrations of mitochondria showed a positive dose-responsive effect on the SH-SY5Y cells treated with the neurotoxin MPP^+^, on acetaminophen-treated hepatocytes and on hippocampal neurons upon injury [16,17,24,69]. However, such a correlation failed in studies of the protective effects on axonal degeneration in nerve explants [69], as well as in vivo studies of the effect of mitochondria on ischemic limbs [70] and injured spinal cord [40]. These results may, nonetheless, be due to the use of saturating amounts of mitochondria in those experiments.

An additional matter refers to the course of mitotherapy. Most publications thus far report a single administration of mitochondria leading to positive results. However, mice with nonalcoholic liver disease chronically treated with mitochondria showed an improvement in markers of damage and a reversal of the injury phenotype [15], suggesting that a chronic treatment with purified mitochondria may be beneficial. This choice will depend on the condition to be treated, as well as further studies to evaluate both the positive and negative effects of an administration of mitochondria.

The mechanisms of the beneficial effects of mitotherapy are yet to be fully understood, inclusive of its eventual specificity. Since many medical conditions involve mitochondrial distress as either cause or consequence, it is usually assumed that adding new mitochondria to the system would rescue the organelle’s related mechanisms. Such a premise explains the prevalence in the literature of targeting the modulation of bioenergetic parameters such as mitochondrial matrix enzymes, NADH, ATP, membrane potential, content of ROS, oxidative stress markers and antioxidant enzymes [7,8,14,16,22,27,29,30,31,40,71]. Indeed, we showed that mitotherapy increased mitochondrial metabolism in the whole retina in the absence of injury [41]. Interestingly, an optic nerve crush led to an increase in retina respiration in untreated rats, which was blocked by intravitreally injected mitochondria, maybe providing the means to keep energy optimized, while hampering harmful signaling.

Notwithstanding, other studies reported results less obviously linked to mitochondrial health. Reduced markers of apoptosis are a common effect of mitochondrial transplantation [7,8,11,14,16,18,21,22,39,71], along with decreased inflammatory cytokines and cell infiltration [8,11,17,30,39]. Free mitochondria or their components have been generally recognized as proinflammatory signals related to DAMPs [72], consistent with proinflammatory modulation [21,40]. Several molecules derived from mitochondria have been recognized as proinflammatory activators such as free mitochondrial DNA (mtDNA), ATP, succinate, N-formylated peptides (N-FP), mitochondrial transcription factor (TFAM) and cardiolipin. In many disorders, these have been shown to be released from damaged mitochondria within dying cells. Some were shown to exert their proinflammatory activities through specific receptors, for instance, mtDNA activates Toll-like receptors 9 (TLR9), ATP signals through the purinergic receptors P2XR and P2YR, N-FP activates formyl peptide receptors (FPR). Thus, mitotherapy becomes a paradigm, since it theoretically would introduce signals that could induce hyperinflammation. However, the current literature advocates a non-inflammatory activity in mitotherapy, such as a reduction in neutrophil infiltration in transplanted hearts [11], reduced reactive astrogliosis at the boundary of ischemic brain area [22], reduced neuroinflammation markers in a model of depression-like behaviors [30], reduced gliosis in a mice model of Alzheimer’s disease [29] and reduced cytokines and inflammatory cell markers in spinal cord ischemic injury [40]. One explanation for these seemingly contradictory activities might be the localization and concentration of the respective molecules. When described as DAMPs and in their pathophysiological context, the mitochondrial contents are out of their physiological compartment, derived from the damaged mitochondria of suffering cells, and are administered directly into the experimental model, thus acting as a proinflammatory signal. In the context of mitotherapy, however, these molecules are compartmentalized and/or in exceptionally low concentrations. For example, MtDNA and TFAM should only be found in the mitochondrial matrix, hidden from TLR9, just as cardiolipin, which is located in the mitochondrial inner membrane and not exposed to the environment. This also applies to N-FP, while succinate and ATP likely have their concentrations diluted in the process of organelle purification. It is also conceivable that those anti-inflammatory effects may be an indirect effect of mitotherapy rather than a direct modulation of inflammatory response since the therapy reduced necrosis, thus reducing the inflammatory signaling induced by cell death.

To better understand the role of immunity in the effects of mitochondrial transplantation, either syngeneic or allogeneic mitochondria were intraperitoneally injected to test for reactivity and tissue recognition using skin grafts and found neither an inflammatory response nor an induction of IgM production, which favors both the safety and usability of mitochondria transplantation [46]. Overall, mitotherapy seems to protect cells from death by regulating mitochondrial bioenergetics and reducing the inflammatory response.

In line with the cellular rescuing effect of mitotherapy, several studies reported the functional recovery of damaged tissues. In the lung, mitotherapy successfully recovered organ performance to sham levels after ischemia [18] and promoted artery relaxation to prevent and reverse pulmonary hypertension [17]. Further, mitotherapy reduced markers of liver damage after ischemia-reperfusion, in nonalcoholic fat liver diseases and in acetaminophen-induced injury [14,15,16]. Remarkably, mitotherapy also reduced the tumor proliferation of lung metastatic melanoma, increased mouse lifespan [19] and enhanced the radiosensitivity of glioma in vivo [58]. A series of studies of heart ischemia-reperfusion induced injury demonstrated the recovery of functional parameters and heart performance measurements along with a reduced size of the infarct area [8,10,11,12,13,51]. Moreover, the same group reported that mitochondria transplantation successfully recovered skeletal muscle from ischemia reperfusion after 24 h, by reducing infarct size and recovering hindlimb function to levels similar to the sham group.

In addition to the advances in several experimental models, mitotherapy has also advanced toward clinical application. Pediatric patients who required central extracorporeal membrane oxygenation support for ischemia-reperfusion-associated myocardial dysfunction after a cardiac surgical procedure were transplanted with autologous isolated mitochondria. Importantly, patients did not develop short-term complications, such as arrhythmia, and four of the five subjects had an improved ventricular function [72]. Despite the urgency of a randomized clinical trial, these data inaugurate the first clinical application of mitotherapy. However, it is not clear whether specific indications support a mitochondrial therapy approach. Theoretically, any disease that is either caused by impaired mitochondrial function or leads to its dysfunction as a consequence of a first insult, might benefit from mitotherapy as mitochondria replenishing. Acute situations, such as an infarct, should benefit from a rapid, single bolus of healthy mitochondria to suppress mitochondrial damage and the consequent cell death, while other pathologies that show a slower but chronic mitochondrial dysfunction may require a persistent administration of the organelle. Care should be taken, and experiments are needed to clarify the limits to continued mitotherapy, in order to avoid the accumulation of free mitochondria and its transformation into DAMPs.

In summary, despite the limited understanding of the mechanisms of mitotherapy, evidence is strong that exogenous mitochondria can help prevent damage, promote functional tissue recovery in several in vivo models of biological disorders, and may, therefore, be applicable in future clinical trials.

## 9. Conclusions

Mitotherapy holds promise from a therapeutic point of view, since most of the studies of diverse diseases and conditions showed positive results. However, important limitations still need to be overcome, such as those raised by the group of Brian O’Rourke, who claimed that mitochondria might not survive the toxic levels of the extracellular calcium concentration [73,74]. Although experiments show that high levels of calcium lead to mitochondrial toxicity and the opening of a permeability transition pore [75], free viable mitochondria have been found in extracellular media such as plasma [76] and cerebrospinal fluid [68]. Additionally, the stability of mitochondria in serum was tested for up to 4 h and the mitochondria remained functional. Still, this issue must be further assessed.

As pointed out, since mitochondria can be easily harvested from cells in culture, clinicians may benefit from dedicated cell culture centers capable of providing fresh mitochondria isolated by a uniform protocol when autologous transplantation is not possible. Further studies are required to address the possibility of chronic treatment, and clinical trials may be on the horizon, pending every ethical and security issue. Overall, mitotherapy still remains a promising treatment, in need of more information to reach its full potential.

## Figures and Tables

**Table 1:** Mitotherapy in animal models of disease by year of publication.

Study Reference	Experimental Model	Animal	Mito Source	Transplant	Route of Administration	Main Result
McCully et al., 2009 [7]	Heart regional ischemia	New Zealand White rabbits	Left ventricular tissue	Allogeneic	Injection into ischemic region	Enhancement of post-ischemic myocardial function
Masuzawa et al., 2013 [8]	Heart regional ischemia	New Zealand White rabbits	Pectoralis major muscle tissue	Autologous	Injection into ischemic region	Enhancement of post-ischemic myocardial function
Lin et al., 2013 [20]	Partial liver ischemia	Wistar rats	Liver	Allogeneic	Intrasplenic injection	Attenuation of hepatic injury
Cowan et al., 2016 [9]	Heart global ischemia	New Zealand White rabbits	Adult cardiac fibroblast culture	Autologous	Coronary artery injection	Enhancement of post-ischemic myocardial function
Su et al., 2016 [17]	Airway hyperresponsiveness	Sprague Dawley rats	Rat airway epithelial cells	Allogeneic	Intratracheally injection	Attenuation of airway remodeling and inflammatory response
Kaza et al., 2017 [10]	Heart regional ischemia	Yorkshire pigs	Pectoralis major muscle tissue	Autologous	Injection into ischemic region	Enhancement of myocardial cell viability
Fu et al., 2017 [15]	Non-alcoholic fatty liver disease	C57BL/6J mice	HepG2 cells	-	Intravenous injection (tail)	Attenuation of lipid accumulation and oxidative stress
Shi et al., 2018 [16]	Acetaminophen-induced liver injury	C57BL/6J mice	HepG2 cells	-	Intravenous injection (tail)	Attenuation of tissue injury and enhancement of hepatocyte metabolism
Moskowitzova et al., 2019 [11]	Heterotopic heart transplantation	C57BL/6J mice	Gastrocnemius muscle	Syngeneic	Coronary artery injection	Enhancement of graft function and attenuation of necrosis
Fu et al., 2019 [19]	Melanoma lung metastasis	BABL/c mice	Liver	Allogeneic	Intravenous injection (tail)	Retardation of tumor growth and prolonged animal survival
Moskowitzova et al., 2020 [18]	Acute lung ischemia-reperfusion	C57BL/6J mice	Gastrocnemius muscle	Syngeneic	Pulmonary artery injection and nebulization	Improvement of lung mechanics and attenuation of tissue injury
Guariento et al., 2020 [12]	Heart regional ischemia	Yorkshire pigs	Pectoralis major muscle tissue	Autologous	Preischemic coronary artery injection	Enhancement of post-ischemic myocardial function
Doulamis et al., 2020 [13]	Warm global ischemia	Zucker diabetic fatty rats	Pectoralis major muscle tissue or cardiac fibroblasts	Autologous and xenogeneic	Coronary artery injection	Enhancement of post-ischemic myocardial function

**Table 2:** Mitotherapy in the central nervous system animal models.

	Study Reference	Experimental Model	Animal	Mito Source	Transplant	Route of Administration
Stroke	Huang et al., 2016 [21]	MCAO	Sprague Dawley rats	BHK-21 cells	Xenogeneic	Injection into ischemic striatum
	Zhang et al., 2019 [22]	MCAO	Sprague Dawley rats	Pectoralis major muscle	Autologous	Intracerebroventricular injection
Parkinson	Chang et al., 2016 [23]	6-OHDA	Sprague Dawley rats	PC12 cells and human osteosarcoma	Allogeneic and xenogeneic	Medial forebrain bundle injection
	Shi et al., 2017 [24]	MPP^+^	C57BL/6J mice	HepG2 cells	-	Intravenous injection
Schizophrenia	Robicsek et al., 2017 [27]	poly-I:C	Wistar rats	Whole brain	Allogeneic	Prefrontal cortex injection
Alzheimer’s disease	Nitzan et al., 2019 [29]	Amyloid-β brain injection	C57BL/6J mice	HeLa cells	-	Intravenous injection (tail)
Depression	Wang et al., 2019 [30]	LPS	ICR mice	Hippocampus	Allogeneic	Intravenous injection
Aging	Zhao et al., 2020 [31]	Aged mice (18 mo)	BABL/c mice	Liver	Allogeneic	Intravenous injection (tail)
Spinal cord	Gollihue et al., 2018 [40]	Spinal cord injury	Sprague Dawley rats	Soleus muscle	Allogeneic	Mediolateral gray matter of injury site
	Fang et al., 2019 [39]	Spinal cord ischemia	Sprague Dawley rats	Soleus muscle	Allogeneic	Intravenous injection (jugular)
Glaucoma	Nascimento-dos-Santos et al., 2020 [41]	Optic nerve crush	Lister hooded rats	Liver	Allogeneic	Intravitreal injection

Middle Cerebral Artery Occlusion (MCAO); 6-hydroxydopamine (6-OHDA); 1-methyl-4-phenylpyridinium (MPP^+^); Polyriboinosinic-polyribocytidylic acid (poly-I:C); Lipopolysaccharides (LPS).

## Abbreviations

MSCs	mesenchymal stem cells
CNS	central nervous system
MCAO	middle cerebral artery occlusion
ICV	intracerebroventricular
PD	Parkinson’s disease
SZ	schizophrenia
Aβ	amyloid-beta
LPS	lipopolysaccharide
RGC	retinal ganglion cell
BBB	Blood–brain barrier
CytC	cytochrome C
TEM	electron microscopy
DAMPs	damage associated molecular patterns
